# A Rare Case of Nephrotic-Range Proteinuria in Antineutrophil Cytoplasmic Antibodies (ANCA)-Associated Vasculitis

**DOI:** 10.7759/cureus.24889

**Published:** 2022-05-10

**Authors:** Rabih Nasr, Pavitra Balasubramanian, Lauren Desiderio, Mohammed Abdelattif

**Affiliations:** 1 Nephrology, BronxCare Health System, New York City, USA; 2 Internal Medicine, BronxCare Health System, New York City, USA

**Keywords:** rituximab therapy, rituximab, pr3-anca, rapidly proliferative glomerulonephritis, anca associated vasculitis, nephrotic

## Abstract

Granulomatosis with polyangiitis (GPA), or Wegener’s granulomatosis as it was formerly referred to, is an antineutrophil cytoplasmic antibody (ANCA)-associated vasculitis (AAV). GPA is characterized as a necrotizing vasculitis with few or no immune deposits termed pauci-immune deposits, predominantly affecting small and medium arterial vessels, involving the upper and lower respiratory tract as well as glomeruli. Renal manifestations are of critical importance because of the progression that may ensue following onset. Glomerulonephritis (primarily rapidly progressive crescentic glomerulonephritis) is quite common, which eventually leads to chronic kidney disease or end-stage renal disease. Usually, patients with GPA and rapidly progressive glomerulonephritis have an elevated plasma creatinine level and urinalysis revealing dysmorphic hematuria, red cell casts, and sub-nephrotic levels of proteinuria. We present a case of a 44-year-old male whose biopsy demonstrated crescentic glomerulonephritis, pauci-immune type proteinase 3 antineutrophil cytoplasmic antibody (PR3-ANCA) consistent with GPA, as well as profound proteinuria, an atypical manifestation.

## Introduction

The incidence of granulomatosis with polyangiitis (GPA) worldwide is estimated to be 10-20 cases per one million population per year and within the United States, it is estimated to be three cases per one million population [1-2]. Though GPA can be seen in all racial and ethnic groups, it is commonly reported in Caucasians with a peak incidence of 64-75 years of age, with no sex predilection as yet [2-4].

Etiologies as well as pathogenesis surrounding antineutrophil cytoplasmic autoantibody (ANCA)-associated vasculitis (AAV) are not well understood and continue to be studied. It is suggested that there are multifactorial etiologies; genetic factors include but are not limited to the *SERPINA-1* gene that codes for alpha-1 antitrypsin, an endogenous inhibitor of proteinase 3 (PR3) may be involved [5]. Microbes such as *Staphylococcus aureus*, though with limited supportive data, may have a role. Drugs containing thiol and hydralazine compounds may be implicated in ANCA seroconversion. Exposures such as silica dust and cigarette smoking may facilitate the disease course. 

GPA is an AAV, in which ANCA plays a critical role in the inflammatory process and pathogenesis. ANCA may be directed against autoantigens PR3 or myeloperoxidase, referred to as PR3-ANCA and MPO-ANCA, respectively. GPA is primarily associated with PR3-ANCA involving a pathogenesis process in which membrane-associated PR3 on neutrophils crosslinks or binds to fragment crystallizable (Fc) receptors, resulting in neutrophil-endothelial cell interactions and microvascular injury; accordingly, when PR3-ANCA reacts with PR3, an enzyme in neutrophil granulocytes, neutrophil activation, adherence to vascular endothelium, and degranulation occurs causing inflammation and endothelial damage.

Indirect immunofluorescence on ethanol-fixed neutrophils yields two major fluoroscopic patterns of AAV. Diffuse cytoplasmic staining or C-ANCA, as well as perinuclear/nuclear staining ANCA (P-ANCA), can be appreciated. Detection of ANCA has a sensitivity of 66% and a specificity of 98% for GPA and is present in 80-90% of patients with active multisystem disease [6].

Regarding classification and diagnostic criteria, the International Chapel Hill Consensus Conference (CHCC) is most commonly cited; CHCC defines GPA as a necrotizing vasculitis with few or no immune deposits predominantly affecting small vessels, involving the upper and lower respiratory tract as well as glomeruli [1-2].

Common clinical presentation of AAV includes fever, fatigue, weight loss, arthralgias, rhinosinusitis, cough, dyspnea, and urinary abnormalities, with or without neurologic manifestations, that following onset may progress slowly over months or severely rapid over days. ENT symptoms include nasal crusting/discharge, sinusitis, earache, otorrhea, oral and/or nasal ulcers, and polychondritis. Development of conductive and/or sensorineural hearing loss may ensue. Saddle nose deformity due to destruction of bone and cartilage is typical in GPA.

Lower respiratory tract involvement in which airways and pulmonary parenchyma have the disease may cause symptoms of hoarseness, dyspnea, cough, hemoptysis, wheezing, and pleuritic chest pain. Pulmonary arterial hypertension or pulmonary fibrosis may develop [6]. Chest radiograph findings may reveal nodules, patchy or diffuse opacities and pulmonary infiltrates, and hilar adenopathy. Cases have been reported in which extra-thoracic tumor-like masses were discovered as well [7].

According to the National Institute of Health (NIH), 18% of patients had evidence of glomerulonephritis upon presentation, and, more significantly, within two years of disease onset, 77-85% of patients had developed glomerulonephritis. Asymptomatic hematuria, elevated serum creatinine with hematuria and cellular casts, subnephrotic proteinuria, and rapidly progressive glomerulonephritis, are some renal manifestations. Pauci-immune glomerulonephritis in which few or no immune deposits in the glomeruli electron microscopy and immunofluorescence is associated with AAV.

Diagnosis should be established through biopsy and findings correlated with the severity of clinical presentation; renal histology may reveal findings extending from mild focal and segmental glomerulonephritis to diffuse necrotizing and crescentic glomerulonephritis [8].

## Case presentation

Our patient is a 44-year-old male who presented to the ED because of ear pain and dyspnea on exertion as well as fatigue. His past medical history was remarkable for type II diabetes mellitus for which he was on metformin 1000mg and hypertension. He denied a history of tobacco, alcohol, and illicit drug use.

**Figure 1:**
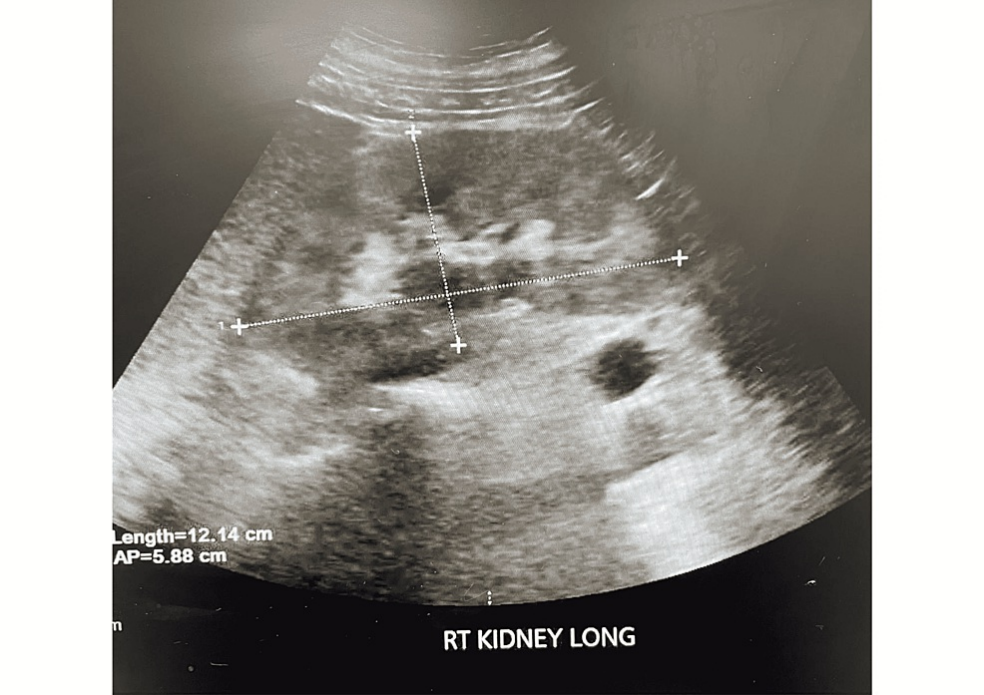
Ultrasound of right kidney showing diffuse increased echogenicity of renal parenchyma consistent with chronic renal disease.

**Figure 2:**
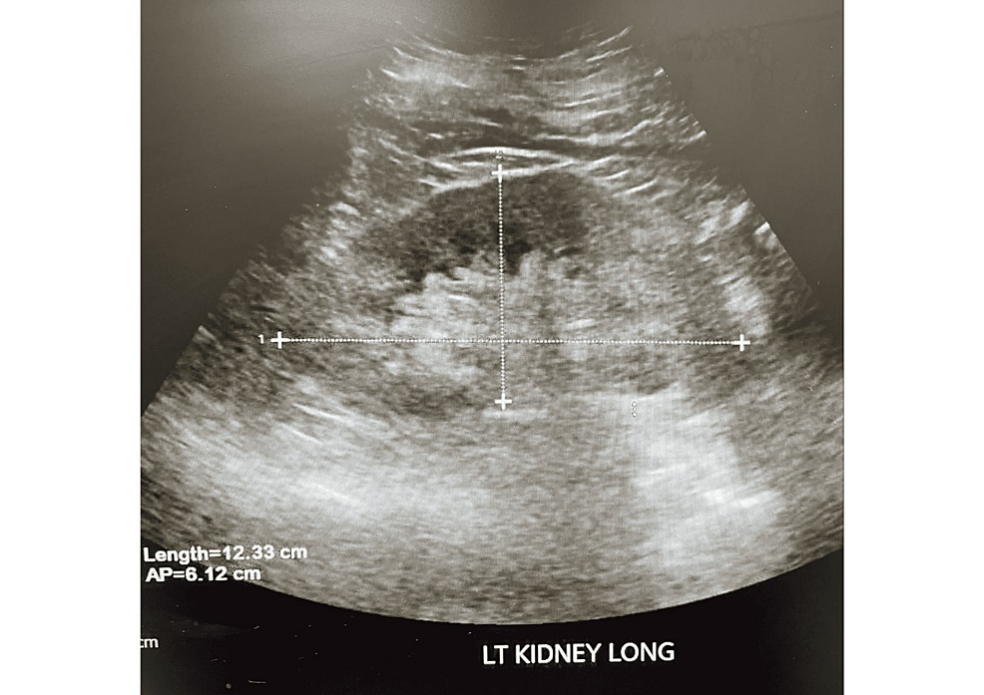
Ultrasound of left kidney showing diffuse increased echogenicity of renal parenchyma consistent with chronic renal disease.

Upon admission our patient was found to have a hemoglobin (HGB) of 6.8 g/dL, hematocrit (HCT) of 21.2 %, WBC count of 11.4k/uL, serum creatinine of 4.7 mg/dL, serum blood urea nitrogen (BUN) of 63 mg/dL, and glomerular filtration rate (GFR) of 10.85 mL/min/1.73 m2. Urinalysis demonstrated pronounced proteinuria of 372mg/dl and moderate blood with few RBCs. The urine protein to creatinine ratio was 7.8mg/mg and the urine microalbumin to creatinine ratio was 268 mg/gm. Erythrocyte sedimentation rate (ESR) was 130 mm/hr and serum c-reactive protein (CRP) was 17.78 mg/L. C3 was elevated with a value of 178 mg/dL and C4 was within the normal range. Immunofixation of urine and serum demonstrated a faint IgG-Kappa monoclonal protein band, consistent with findings a year prior during which serum-free lambda was 126.6 mg/L, serum-free kappa was 280 mg/L, and free kappa lambda light chains ratio was 2.21. Such findings with anemia raised suspicion for multiple myeloma, though calcium levels were within normal range, the x-ray skeletal survey was unremarkable except for a lucency in the right hip. C-ANCA screen was positive at a value of 54 mg/dL. Abdominal and pelvic ultrasound revealed diffuse increased echogenicity of bilateral renal parenchyma consistent with chronic renal disease (Figures 1, 2). 

**Figure 3:**
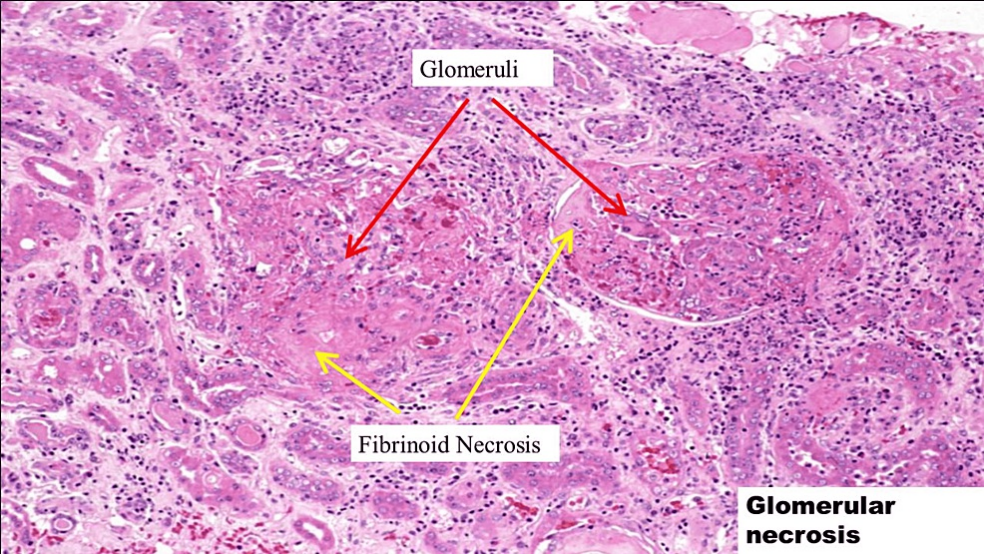
Gram stain showing focal necrosis and diffuse crescentic glomerulonephritis.

The patient underwent a CT scan-guided renal biopsy, which showed focal necrotizing and diffuse crescentic glomerulonephritis (as seen in Figure 3), pauci-immune type PR3-ANCA. It also showed severe tubular atrophy and interstitial fibrosis as well as mild arteriosclerosis. Immunofluorescence showed nonspecific segmental glomerular tuft staining for IgM, C3, and C1, consistent with trapping in areas of sclerosis. No evidence of an immune complex of paraprotein deposition was observed. Overall histologic findings were consistent with PR3-ANCA vasculitis. 

**Figure 4:**
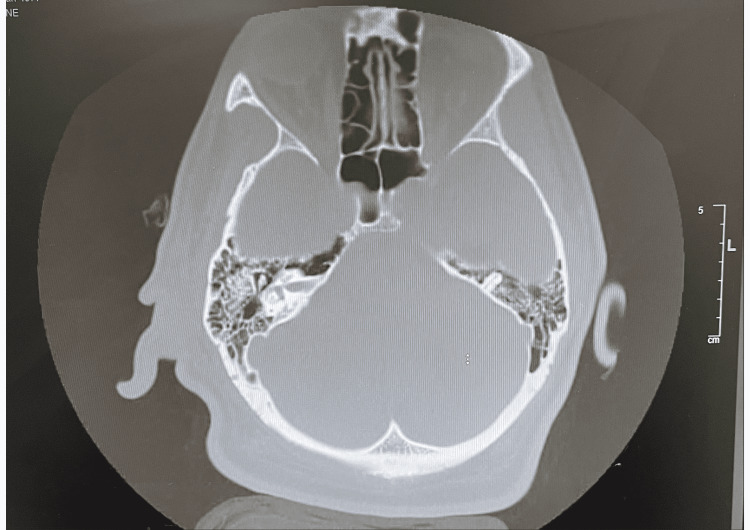
CT ears without contrast showed gross soft tissue edema about the pinna of the right ear and within subcutaneous fat at the base.

**Figure 5:**
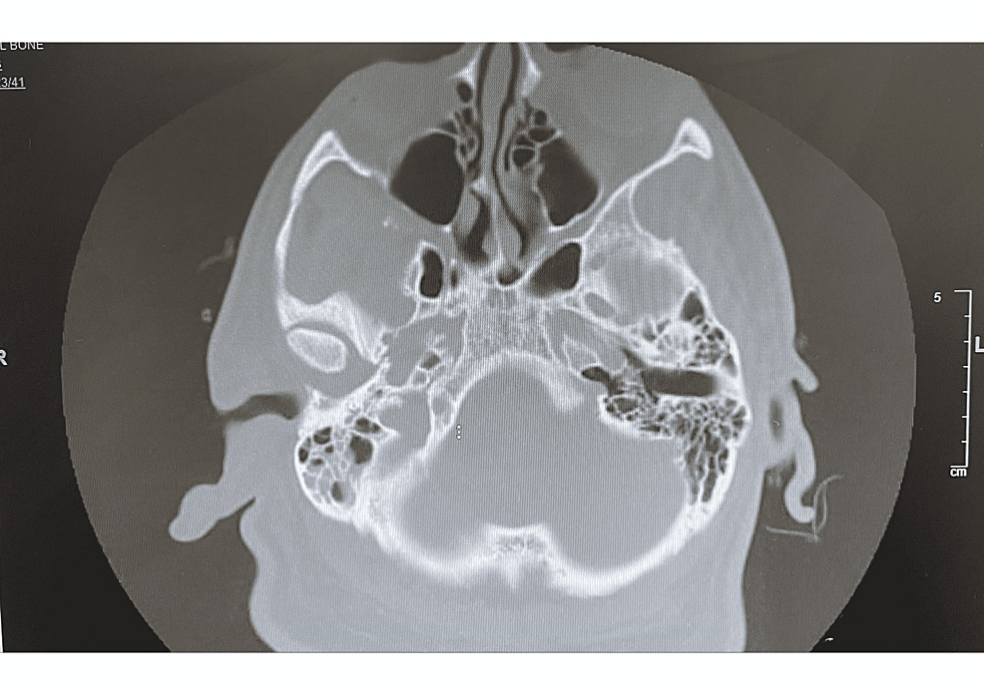
CT ears without contrast showed fluid-filled inferior right mastoid air cells to support the case presentation.

CT of ears without contrast was remarkable for gross soft tissue edema about the pinna of the right ear and within subcutaneous fat at the base and inferior right mastoid air cells were fluid-filled (Figures 4, 5). Findings were concerning for right mastoiditis with overlying pinna perichondritis. Management with cefepime IV, vancomycin IV, and diphenhydramine was done.

Initial induction therapy for a patient with GPA without concomitant anti-glomerular basement membrane (anti-GBM) disease active glomerulonephritis was begun. A rituximab regimen of 1g followed by another 1g dose in 14 days was administered with high-dose IV Pulse methylprednisolone (1000mg for three days followed by a taper).

## Discussion

**Figure 6:**
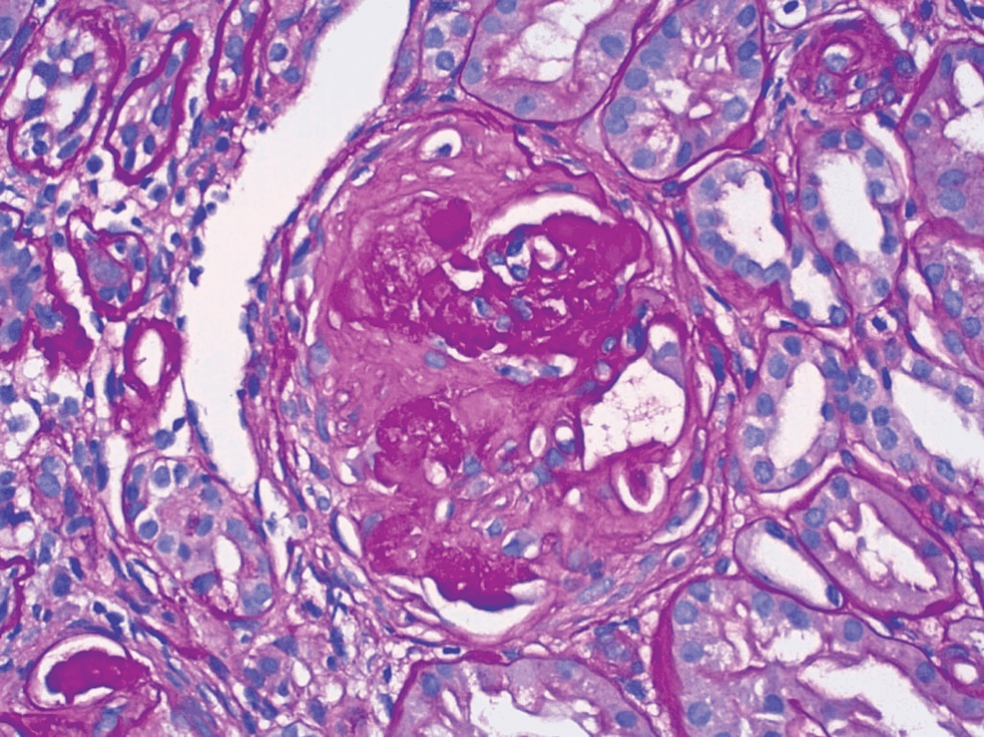
Gram stain showing crescentic glomerulonephritis

Here we have a case of rapidly worsening renal failure associated with chondritis, nephrotic-range proteinuria, and PR3-ANCA on serology. The patient on presentation had no symptoms of edema, decreased urine output, or hematuria. The urinalysis in glomerulonephritis typically reveals dysmorphic hematuria, RBCs, and other casts. However, our patient demonstrated only rare RBCs on the urinalysis. The marked reduction in GFR usually limits the rate of protein filtration leading to a sub-nephrotic range of proteinuria. Nephrotic syndrome is unusual and is most likely to occur in patients with less severe renal insufficiency. The presence of nephrotic-range proteinuria in our patient with a protein to creatinine ratio of 7.8mg/mg creatinine and microalbuminuria with a urine microalbumin to creatinine ratio of 268 mg/gm stood out in the investigations. The findings in the serum and urine immunofixation and the nephrotic-range proteinuria initially raised suspicion of multiple myeloma. But the C-ANCA screen coming back positive with a value of 54mg/dl brought the diagnosis of AAV into the differential. The renal biopsy showing focal necrotizing and diffuse crescentic glomerulonephritis (as seen in Figure 6), pauci-immune type, helped confirm the diagnosis.

Heavy proteinuria in ANCA-associated glomerulonephritis (ANCA GN) is usually seen with immune complex deposits in renal biopsy. Nephrotic ANCA GN with pauci-immune deposits is rare and has not been studied in depth. In a study by Xu et al., it was demonstrated that pauci-immune ANCA GN with nephrotic proteinuria was associated with crescents seen in histopathology, an increased incidence of AKI, gross hematuria, and poorer renal prognosis despite the proteinuria having a good sensitivity to therapeutic modalities [9].

There has been no clear conclusion regarding the relationship between proteinuria and renal prognosis of AAV in previous studies. Pauci-immune necrotizing crescentic glomerulonephritis is the most common histopathological type of ANCA GN, but nephrotic-range proteinuria is rarely reported in these cases. What is commonly noted is that ANCA GN, especially with high amounts of proteinuria, is often seen superimposed on, or in association with, other glomerular disease processes characterized by glomerular immune deposits. In these cases, the increased proteinuria may be due to the synergetic effects of the multiple coexisting diseases. [10-12]

Even though massive proteinuria is usually the initial disorder in nephrotic syndrome (NS), the exact pathogenesis of hypoalbuminemia in NS is not clear, because it is not always associated with nephrotic-range proteinuria [13]. In their study, Xu et al. found that there was no difference in the serum albumin levels of ANCA GN patients with and without nephrotic-range proteinuria, which indicates that proteinuria may not induce hypoalbuminemia in AAV. Some possible explanations are: (1) ANCA GN is a form of severe proliferative nephritis and concomitant tubulointerstitial damage may cause a non-selective excretion of proteins, (2) hypoalbuminemia may be due to massive proteinuria along with the combined effects of inflammation and inadequate nutritional intake in patients with inflammatory disease, and (3) one of the factors influencing hypoalbuminemia in nephrotic syndrome could be increased capillary permeability [14].

## Conclusions

The rapid decline in renal function and quick progression to end-stage renal disease (ESRD) in our patient with AAV is worth noting. This case highlights the importance of clinicians being vigilant and prompt in the diagnosis and management of patients with AAV with nephrotic-range proteinuria to preserve renal function and delay the onset of ESRD.
